# Evaluation of Depression, Anxiety, Stress, and Burnout Levels Among Emergency and Intensive Care Unit Professionals

**DOI:** 10.1192/j.eurpsy.2025.485

**Published:** 2025-08-26

**Authors:** S. Kukurt, G. Dokuz, O. Kilic, Z. L. Onat, M. Combas, I. Kirpinar

**Affiliations:** 1 Psychiatry, Bezmialem Vakif University, Istanbul, Türkiye

## Abstract

**Introduction:**

Emergency departments (ED) and intensive care units (ICU) are high-stress environments where healthcare professionals are continuously exposed to critical situations. This results in substantial mental health burdens, leading to increased levels of depression, anxiety, stress, and burnout. These issues negatively affect both the well-being of professionals and patient care.

**Objectives:**

This study aims to evaluate the prevalence and severity of depression, anxiety, stress, and burnout among ED and ICU professionals. The goal is to understand mental health issues and identify contributing factors to improve prevention and support systems.

**Methods:**

A total of 242 healthcare professionals from Bezmialem Vakif University Hospital were included: 120 ICU staff (80 females, 40 males), 66 ED personnel (42 females, 24 males), and 56 office personnel (38 females, 18 males) serving as a control group. The study employed validated instruments: the Beck Depression Inventory, the Beck Anxiety Inventory, Perceived Stress Scale, and Maslach Burnout Inventory. Non-parametric tests (Kruskal-Wallis H and Chi-Square) were used due to non-normal data distribution, with pairwise comparisons adjusted using Bonferroni correction. The significance level was set at p < 0.05.

**Results:**

The results showed significant differences in age, depression scores, stress levels, and burnout indicators between the ICU, ED, and control groups. ICU and ED staff reported significantly higher depression scores compared to the control group (p < 0.001). Stress levels were also significantly elevated in ICU workers compared to office personnel (p = 0.001). Burnout indicators were notably higher in ICU professionals (p = 0.011). Conversely, no significant differences were observed in anxiety scores, emotional exhaustion, and hours of sleep.

**Table 1:** Summary of Key Results

Variable	ICU Personnel (n = 120)	ED Personnel (n = 66)	Office Personnel (Control, n = 56)	p-value	Significant Difference
Beck Depression Score (Median)	10	10	6	<0.001	Yes
Beck Anxiety Score (Median)	8	7	6	0.064	No
Perceived Stress Score (Median)	25.5	23	23	0.001	Yes (ICU vs. Office)
Maslach Depersonalisation Score (Median)	23.5	21	21	0.011	Yes (ICU vs. Office)
Maslach Personal Accomplishment Score (Median)	23.5	22	21	0.034	Yes (ICU vs. ED)
Maslach Emotional Exhaustion Score (Median)	22	21	21	0.064	No
Working Hours (Median)	60	55	40	<0.001	Yes
Living with Family/Partner/Alone (%)	65% / 25% / 10%	60% / 30% / 10%	70% / 20% / 10%	0.232	No
Satisfaction with Job (%)	65%	70%	80%	0.147	No

**Conclusions:**

The findings underscore the necessity for targeted interventions to reduce psychological distress among ED and ICU professionals. Implementing support systems, promoting work-life balance, and improving mental health resources can significantly alleviate the mental burden on these professionals, thereby enhancing both their well-being and patient care quality.

**Disclosure of Interest:**

None Declared

